# On-Demand Hydrogen Generation by the Hydrolysis of Ball-Milled Aluminum–Bismuth–Zinc Composites

**DOI:** 10.3390/ma15031197

**Published:** 2022-02-04

**Authors:** Jamey Davies, Stephanus P. du Preez, Dmitri G. Bessarabov

**Affiliations:** Hydrogen South Africa (HySA) Infrastructure, Faculty of Engineering, North-West University (NWU), Private Bag X6001, Potchefstroom 2520, South Africa; daviesjamey@gmail.com (J.D.); dmitri.bessarabov@nwu.ac.za (D.G.B.)

**Keywords:** aluminum, mechanochemical activation, ball milling, bismuth (Bi), zinc (Zn), hydrolysis, hydrogen generation

## Abstract

In this investigation, ternary Al-Bi-Zn composites were prepared through mechanochemical activation to determine the combined effects of low-cost Bi and Zn on the morphology change and reactivity of the Al composite during the hydrolysis reaction. Specifically, Zn was considered as a means to slow the hydrogen generation rate while preserving a high hydrogen yield. A steady hydrogen generation rate is preferred when coupled with a proton exchange membrane fuel cell (PEMFC). Scanning electron microscopy (SEM) analysis indicated that Bi and Zn were distributed relatively uniformly in Al particles. By doing so, galvanic coupling between anodic Al and the cathodic Bi/Zn sustains the hydrolysis reaction until the entire Al particle is consumed. X-ray diffraction analysis (XRD) showed no intermetallic phases between Al, Bi, and/or Zn formed. A composite containing 7.5 wt% Bi and 2.5 wt% Zn had a hydrogen yield of 99.5%, which was completed after approximately 2300 s. It was further found that the water quality used during hydrolysis could further slow the hydrogen generation rate.

## 1. Introduction

Progression in fuel cell technology and the necessity for environmentally friendly sustainable energy carriers motivates the development of more efficient hydrogen production methods. Although hydrogen is abundant on earth, it does not occur naturally in its pure form and has to be processed to be converted to an energy carrier [1,2]. Numerous processes are employed to produce hydrogen from various source materials, e.g., electrochemical, [3] photo-electrochemical, [4,5] photo-chemical, [6] photo-biological, [4,7] photo-catalytical, [4,8] partial hydrocarbons oxidation, [9] photo-thermochemical, [10] and niche and nanoparticle-assisted biological methods [11,12,13,14,15,16].

Partial hydrocarbon oxidation and photo-thermochemical methods are mainly used to produce hydrogen. In both processes, carbon dioxide (CO_2_) and small amounts of carbon monoxide (CO) are formed, which adds to the accumulation of climate-changing gases in the atmosphere [2,17]. Hydrogen can only be considered green if it is produced from a renewable source (e.g., water) using a renewable energy source. However, this approach contains its difficulties, such as the intermittent availability of solar and wind energy [18].

Producing hydrogen in a renewable and eco-friendly manner is difficult, and more so, the storage thereof. Hydrogen storage is complicated due to its low gaseous density of 0.09 kg/m^3^ and a relatively high liquidous density of 70.9 kg/m^3^ [18]. Furthermore, hydrogen is flammable over a wide concentration range of 4–75 vol% and has low ignition energy of 0.02–0.03 mJ. A static electricity discharge or agitation of compressed or liquid hydrogen can cause hydrogen to ignite [19]. Out of all known energy carriers, hydrogen has the highest mass-specific energy density, with lower heating value (LHV) and higher heating value (HHV) of 120 and 142 MJ/kg, respectively [18,20]. Finding a method that releases hydrogen when required will reduce the struggles regarding its storage. Generated hydrogen can be stored and used in various ways, e.g., domestic applications (cooking and spatial heating), [21,22,23,24,25,26,27] power generation and transportation sectors (fuel cells, internal combustion engines), [28,29,30] petrochemical industry, [31] and in ammonia and methanol production [32,33].

On-demand hydrogen generation via the hydrolysis of lightweight metals with neutral pH water is an attractive approach. To ensure this hydrogen generation method is employed to its fullest potential, it is necessary to identify an appropriate metal. Several studies have shown that aluminum (Al) is currently the most suitable candidate, due to its abundance, its potential to be fully recycled, and its light weight [34]. Furthermore, the complete hydrolysis of Al yields up to 1.36 L hydrogen per gram of metal under standard ambient conditions [35]. Al hydroxides, hydrogen, and heat are formed during the spontaneous and exothermic Al hydrolysis. The hydrolysis reaction of Al can be expressed as the following:

2Al + 6H_2_O → 2Al(OH)_3_ + 3H_2_ + 16.3 MJ/kg Al (1)

2Al + 4H_2_O → 2AlOOH + 3H_2_ + 15.5 MJ/kg Al (2)

Not only is the Al hydrolysis reaction favorable for generating hydrogen, but the produced Al hydroxides (gelatinous, water-insoluble material) can be converted into alumina (Al_2_O_3_). The Al_2_O_3_ can subsequently be recycled to metallic Al via the energy-intensive Hall–Héroult process [35,36]. More so, various water qualities, e.g., deionized, filtered, tap, seawater, and urine, can be used for the hydrolysis of Al [37,38,39,40].

It is well understood that a thin and coherent Al_2_O_3_ layer forms on the surface of Al particles, which prevents the interaction between the underlying Al metal and water. This passivation layer can be removed in an alkaline [41] or acid [42] solution. However, these solutions are corrosive, and pH values of <5 and >9 are required, as Al passivates at a pH of 5–9 [43]. Another approach is to remove the oxide layer via amalgamation, i.e., the formation of Al_2_O_3_ is inhibited/the formed Al_2_O_3_ layer is disrupted by mercury (Hg)- or gallium (Ga)-based composites. However, this method is complicated by the toxic nature of Hg and the expensiveness of Ga [44]. Thermal or mechanochemical processing of Al with various salts, metals, metal oxides, and combinations thereof are attractive techniques to remove the protective Al_2_O_3_ layer, e.g., Al-x (x = combinations of In, Sn, Ga, Bi, Zn, Li, Fe, Mg, Cu, Ti) [38,45,46,47,48,49,50], Al-BaCl_2_, [51] Al-KCl, [52] Al-BiOCl, [53] Al-NaBH_4_, [54] Al-NaMgH_3_, Al-Al(OH)_3_, [55] Al-Fe, [56] Al-Bi-hydride/various salts, [57] Al-Ni-NaCl, [58] Al-Bi-C, [59] Al-InCl_3_-(Ni-Bi-B), [60] Al-Zn-B [61], Al-Ga-In-SnCl_2_, [62] Al-NaMgH_3_-Bi-Li_3_AlH_6_, [63] Al-Ga-In-Sn-KCl, [64], and processed, technical-grade aluminum, Al-6061 (containing Mg and Si) [65].

Metals with a more positive electrode potential than Al (1.662 V) are generally considered as activation metals. However, such activation metals must accelerate the structural degradation of Al during the mechanochemical process (ball milling), which results in the relatively uniform distribution of these metals throughout Al particles. This uniform distribution of the activation metals leads to the formation of numerous galvanic cells between Al (anodic) and activation metals (cathodic) during the hydrolysis reaction. These micro-galvanic cells provide the Al particles with continuous hydrolysis activity.

Although both mechanochemical and thermal processing is used to generate Al composites, mechanochemical activation is considered a less energy-intensive approach when compared to thermal processing. Mechanical activation allows starting materials to be converted to micro-sized particles at ambient, or close to ambient, conditions. More so, mechanochemical activation enables the formation of composites void of contaminants as a result of unwanted side reactions [66].

Hydrogen generated through the hydrolysis of mechanochemically processed Al can be combined with proton exchange membrane fuel cells (PEMFCs) where the chemical energy of hydrogen is converted into an electrical current. Hydrogen generated through this method will eliminate the presence of Pt-poisoning CO(g). The application of PEMFC can be advantageous, as it is considered to be ideal for mobile energy applications (unmanned aerial vehicles and road vehicles), portable electrical devices with low power demand, and stationary power units [67,68,69].

Nonetheless, the employment of Al composites to generate hydrogen is limited to the laboratory scale due to several reasons: the high expense of activation metals (e.g., Ga-activated composites), the instability of activation metals, toxicity (e.g., Hg-activated composites), and the corrosiveness of activation chemicals (acidic/alkaline solutions).

Furthermore, Al hydrolysis typically proceeds rapidly, which complicates its coupling with PEMFCs. Therefore, the use of Zn as an activation metal to possibly delay the hydrogen generation rate is considered [11,41]. More so, the exclusion of Ga was also important to minimize the cost of Al activation. Though, Wang et al. (2013) and (2016) prepared Ga-containing Al-composites with >99% hydrogen yields [38,70]. Nevertheless, the presence of Ga increases production cost, motivating the employment of less expensive additives such as Bi, Zn, etc.

The use of Bi has been explored as an Al activation metal [71,72,73,74,75]. The combination of Bi with dissimilar activation metals has yet to receive significant attention. A study by Wang et al. (2021) prepared Al-Bi-Zn composites using a gas atomization method. An Al-12 wt% Bi-7 wt% Zn composite achieved a 98% hydrogen yield after 280 min when hydrolyzed in distilled water at 50 °C [50]. In this study, ternary Al-Bi-Zn composites were prepared by ball milling, and their reactivity towards neutral pH water was evaluated. The effects of Bi and Zn on the microstructure of Al were characterized, and any mineralogical interactions between the constituents were determined. A specific objective of this study was to determine the feasibility of employing Zn as an activation metal to retard the release of hydrogen during hydrolysis while preserving the composite reactivity.

## 2. Materials and Methods

### 2.1. Materials

The following materials used in this study were purchased from Sigma-Aldrich (Johannesburg, South Africa): Al powder (<200 mm, 95% purity), Bi granules (>99.9% purity), and Zn powder (<150 mm, >99.8% purity). Hydrolysis procedures were performed using tap water, filtered water, and deionized water (18.2 MΩ cm^−1^ resistivity) produced by a Milli-Q water purification system (Sigma-Aldrich, Johannesburg, South Africa). Some hydrolysis reactions were performed in sodium chloride (NaCl) solutions. The NaCl (99.5%) was supplied by Sigma-Aldrich (Johannesburg, South Africa). Pure nitrogen gas (99.9%; Afrox, Johannesburg, South Africa) was used for all purging procedures.

### 2.2. Aluminum Composite Compositions and Their Mechanochemical Preparation

Ternary composites consisting of a fixed amount of Al (90 wt%, and 95 wt% in one case) and various amounts of Bi and Zn combinations (collectively 10 wt%, and 5 wt% in one case) were prepared. The total amount of material per milling procedure was kept at 5 g. The compositions of the Al composites are given in Table 1.

All the Al composites exhibited in Table 1 were prepared via high-energy mechanochemical activation using the Emax ball mill (Retsch, Düsseldorf, Germany) under a nitrogen atmosphere. Each composite constituent was weighed (as specified in Table 1) and placed in a 250 mL stainless steel milling jar with 5 mm stainless steel milling balls. The milling balls to material mass ratio was 30:1 (defined as 30 g of milling balls per 1 g of composite). Subsequently, the milling jar was sealed with an aerated stainless-steel lid and purged with nitrogen before milling. All composites were milled for 30 min at 1500 rpm. After milling, the ball-milled samples were allowed to cool to room temperature while being kept in the sealed milling jars. To eliminate any possibility of unwanted atmospheric oxidation, hydrolysis reactions were performed immediately after recovering the composites from the milling jars. Each composite was ball milled in duplicate. Samples that exhibited repeatability in terms of physical change and size reduction after 30 min of ball milling were examined for a third repetition. Some Al composites formed large particles after 30 min of ball milling; these composites presented limited to no reactivity and were excluded from further experimentation.

### 2.3. Hydrolysis Set-Up and Hydrogen Measurements

The reactivity of each composite was determined by performing hydrolysis reactions in a 250 mL three-neck flask under standard ambient conditions. The flask openings were used for hydrogen escape, hydrolysis solution addition, and as a thermocouple port. The produced hydrogen passed through a gas drier containing a combination of Drierite^TM^ (Sigma-Aldrich, Johannesburg, South Africa) to remove water vapor before hydrogen measurements. The volume of generated hydrogen was measured using a digital gas mass flow meter (Model GM-32654-12; Cole-Parmer, Johannesburg, South Africa). Hydrogen measurements were performed using 1 g of composite and 50 mL of the reaction solution unless specified otherwise. The reaction solutions were left unagitated (stirring or ultrasonication) during all hydrolysis reactions.

The hydrogen generated during the hydrolysis reaction was expressed as a yield %, defined as the volume of generated hydrogen over the theoretical volume of obtainable hydrogen, assuming complete hydrolysis of the Al content. By applying the ideal gas law, approximately 1360 mL of hydrogen per gram of Al is obtainable after complete hydrolysis under standard ambient conditions.

### 2.4. Composite Characterization

X-ray diffraction (XRD) measurements were carried out using a Röntgen diffraction system (PW3040/60 X’Pert Pro, Malvern Panalytical, Malvern, UK). A back-loading preparation method was applied to determine the crystalline phases present in the al composites and hydrolysis residues. Measurements were performed using a multipurpose X-ray diffractometer D8-Advance from Bruker (Billerica, MA, USA) operated in a continuous θ-θ scan in locked coupled mode with Cu-K_a_ radiation. The sample was mounted in the center of the sample holder on a glass slide and leveled up to the correct height. The measurements ran within a range of 2θ with a typical step size of 0.034°. A position-sensitive detector, Lyn-Eye, was used to record diffraction data at a typical speed of 0.5 s/step, which is equivalent to an effective time of 92 s/step for a scintillation counter. The identification of the detected phases was performed using X’Pert HighScore Plus software (v.5.1, Malvern Panalytical, Malvern, UK).

The morphology and/or chemical characterization of the as-received Al powder and Al composites were performed by using a scanning electron microscope (SEM) equipped with an energy-dispersive X-ray spectrometer. An FEI Quanta 250 FEG scanning electron microscope incorporated with an Oxford X-map energy dispersive X-ray spectrometer system, operating at 7 kV and a working distance of approximately 10 mm, was used (FEI Company, Hillsboro, OR, USA). Samples chosen for investigation were mounted on a sample stub using a carbon-based adhesive tape and coated with a thin layer of carbon of about 3 nm.

SEM micrographs were obtained at various magnifications (µm scale), which is indicated on all micrographs presented. SEM-EDS was employed to determine the distribution of activation metals on the surface.

## 3. Results and Discussion

### 3.1. Effects of Balling on Characteristics of Al Composite Particles

According to Benjamin (1976), several metal composites can be generated by milling the metals together even in the absence of a lubricant. Furthermore, it was indicated that ball mills achieved higher energies during milling than conventional mills, leading to rapid metal composite formation and reducing the effect of initial material particle size on the final composite homogeneity [76].

Three types of mechanochemical activation combinations occur during ball milling: ductile–ductile, ductile–brittle, and brittle–brittle. Al is a ductile metal, whereas Bi and Zn are considered brittle metals under standard ambient conditions [77,78,79].

During ball milling, ductile and brittle metals undergo repetitive plastic deformation, which causes radical changes in particle shape, residual stress, and the redistribution of metal constituents. Several mechanisms occur during ball milling that can explain the radical changes taking place during the ball milling process. At the onset of milling, particles are caught between the impacts of milling equipment, e.g., ball–ball and ball milling chamber, causing the particles to agglomerate due to cold welding. Large, cold-welded particles consist of an unequal distribution of as-received constituents (Al, Bi, and Zn in this case). As ball milling continues, the cold-welded particles undergo plastic deformation until a certain stress-to-strain point is reached. Hereafter, the particles harden due to work hardening. Work-hardened particles are resistive to cold welding and will fracture into smaller particles when caught between impacts. This results in a decrease in the average particle size [79,80].

**Figure 1:**
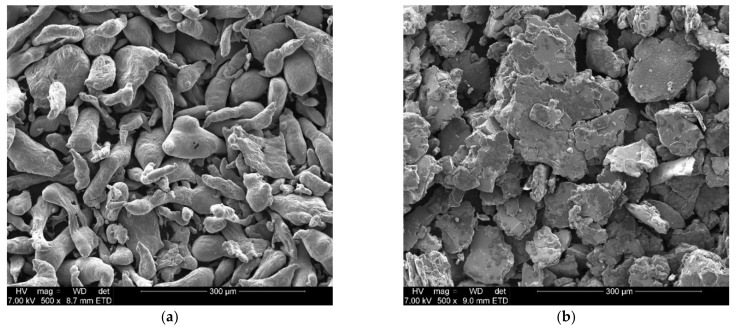
Secondary electron SEM micrographs of as-received Al (**a**) and mechanochemically processed Al-5% Bi-5% Zn composite (**b**) particles.

Figure 1 presents secondary electron SEM micrographs of the as-received Al particle (a) morphology and the Al-5% Bi-5% Zn composite (b) to evaluate the effect of milling on the morphology of Al in the presence of Bi and Zn.

It is evident from Figure 1 that after the mechanochemical process employed here, the as-received Al (a) changed from the initial 100–300 µm, uneven, strand-like morphology to a platelet morphology with a slight decrease in particle size (b). The small reduction in particle size can be explained by the following: during cold welding, the average particle size increases, while during fracturing, the average particle size decreases, except for very small, hardened particles that can resist fracturing; these small particles tend to weld onto larger particles after a state of equilibrium is reached between the rate of cold welding and the rate of fracturing during ball milling. This results in fine and large particle sizes to be converted to intermediate size, which creates a significant change in particle size consisting of both small and large particles, as seen in Figure 1b [76,77,81]. The presence of a steady-state equilibrium between the rate of cold welding and the rate of fracturing was also observed by du Preez et al. (2017) while investigating the hydrolysis of activated Al-In-Bi-Sn composites for hydrogen generation [12].

**Figure 2:**
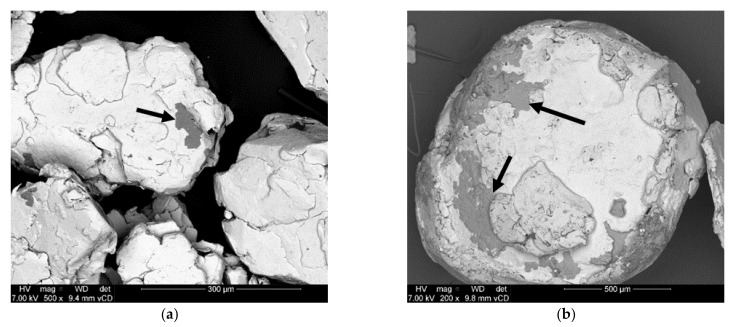
Backscattered electron SEM micrographs of mechanochemically processed Al-2.5% Bi-7.5% Zn (**a**) and Al-2.5% Bi-2.5% Zn composite (**b**) to emphasize the incomplete composite constituency distribution (indicated by arrows).

Figure 2 is included to exemplify the morphological changes of composites that did not undergo complete/appreciable mechanochemical activation during the milling procedure employed here. Micrographs are presented in back-scattered mode to emphasize non-uniform composite constituent distribution (indicated with arrows). The presented composites, Al-2.5% Bi-7.5% Zn and Al-2.5% Bi-2.5% Zn, were selected as they were composed of visually large particles and exhibited no hydrolysis reactivity.

Of the prepared Al-Bi-Zn composites (as indicated in Table 1), the Al-2.5% Bi-7.5% Zn and Al-1% Bi-9% Zn composites had no hydrolysis reactivity. The Al-1% Bi-9% Zn composite adhered to the interior of the milling jar and could not be recovered after the ball milling procedure. Though ball milling of the Al-2.5% Bi-7.5% Zn composite yielded a granular powder, the composite did not exhibit any hydrolysis activity. The observed inactivity of the composite may jointly be ascribed to a decrease in reaction surface (large particles of >300 µm, Figure 2a) and the improper distribution of activation compounds (indicated by the arrows in Figure 2).

The effect of activation metal addition on particle morphology was further emphasized by reducing the Bi and Zn additions from a combined value of 10 wt% to 5 wt% in the Al composite. The resulting composite (i.e., Al-2.5% Bi-2.5% Zn) is shown in Figure 2b. It can be seen that large particles (>1000 um) were obtained, and the presence of gray areas suggests the non-uniform distribution of composite constituency.

Nevertheless, a reduction in the composite particle size does not guarantee that Al will be reactive with water under standard ambient conditions. To ensure Al hydrolyses proceed, it is essential to remove (or at least disrupt) the protective Al_2_O_3_ layer present on the surface of Al particles. The presence of activation metals, e.g., Bi and Zn, on the surface of Al disrupts and prevents the reformation of the O-rich passivation layer as they prevent the interaction between Al and ambient O. Of equal importance is the uniform distribution of activation metals throughout the Al particles. By doing so, numerous micro-galvanic cells between anodic Al and cathodic activation metals are formed, which sustains the Al hydrolysis reaction until the available Al is hydrolyzed.

The formation of micro galvanic cells depends on the standard electrode potentials, i.e., E° (V), of the activation metals, and it should be more positive than the E° (V) of Al (−1.662 V). Considering that the E° of Bi and Zn is 0.308 V and −0.760 V, respectively, galvanic coupling between anodic Al and cathodic Bi and Zn will result in the oxidation of Al^0^ to Al^3+^ and the subsequent ejection of Al^3+^ into the aqueous phase.

Reboul et al. (1984) presented a mechanism where the Al and the activation metals are firstly oxidized by galvanic coupling, as illustrated in Reaction 3 [82]:

Al(M) → Al^3+^ + M^n+^, M = activation metal (3)

The M^n+^ possess a cathodic nature relative to Al, causing these ions to deposit onto the fresh Al surfaces through an electrochemical exchange reaction. The reaction occurs as follows:

Al + M^n+^ → Al^3+^ + M (4)

Reaction 4 shows that Al is oxidized to Al^3+^, enabling it to migrate from the solid matrix into the solution where the hydrolysis reaction proceeds.

**Figure 3:**
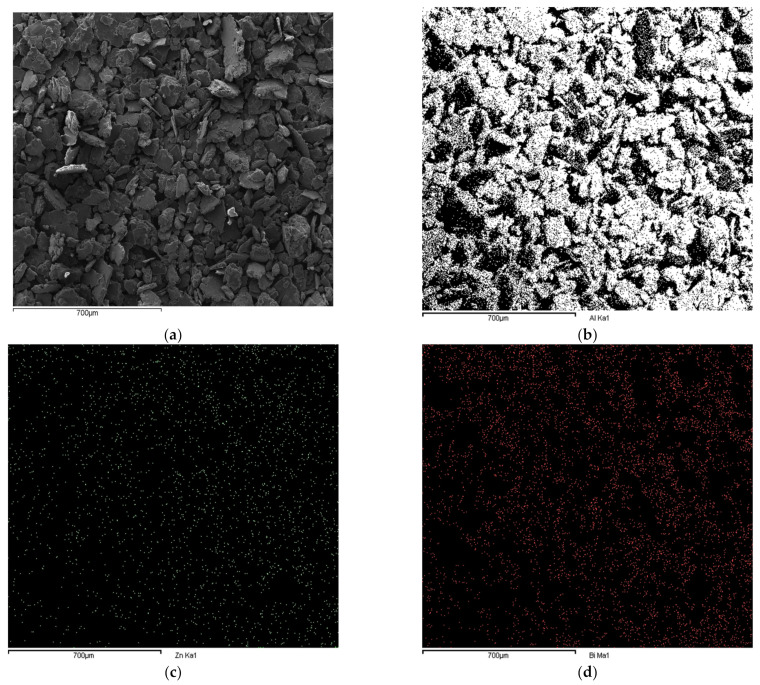
Secondary SEM micrograph of the surface of Al-5% Bi-5% Zn (**a**) and the corresponding EDS mappings for Al (**b**), Bi (**c**), Zn (**d**).

To investigate the distributions of Bi and Zn, EDS mapping was performed on the surface of Al-5% Bi-5% Zn particles (Figure 3).

Figure 3 shows a relatively even distribution of the activation metals (Bi and Zn) on the surface of the particles, indicating that the formation of micro-galvanic cells is expected. Figure 3 further indicates that Bi or Zn does not exist as individual agglomerates or chunks.

Figure 3 only shows the uniform distribution of activation metals on the surface of the particles. Recent studies by du Preez et al. (2018) and (2019) focused on Al’s activation by Sn-In and Bi-Sn, respectively. These studies showed that the respective activation metals, which did not exceed a total of 10 wt%, were distributed relatively uniformly throughout Al particles [11,13,45]. Considering these studies, it can be assumed that the distribution of Bi-Zn will be similar to that of Sn-In and Bi-Sn. More so, considering that Bi has no appreciable solid solubility in Al, while Zn has a solubility of <1 wt%, the inclusion of Bi and Zn within Al particles will likely only be viable via a mechanochemical route [83,84,85].

### 3.2. XRD Analysis of Ternary Al Composites

**Figure 4:**
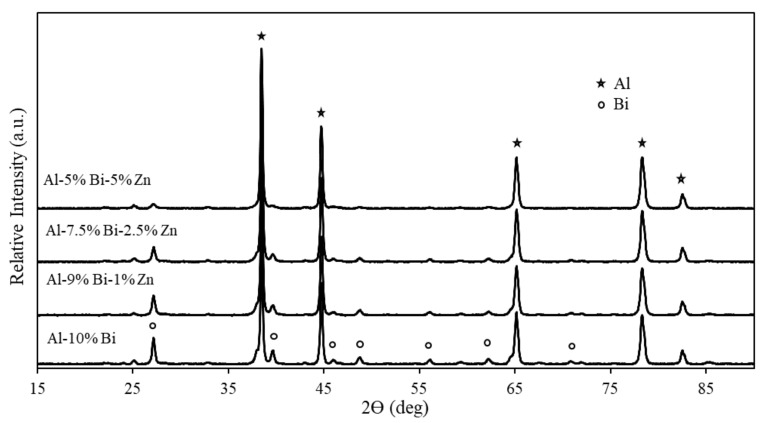
XRD patterns of binary Al-Bi and ternary Al-Bi-Zn composites.

XRD analysis was performed on ternary Al composites to investigate the chemical composition of the Al composites containing various amounts of Bi and Zn. These XRD patterns are presented in Figure 4. The Al composites shown in Figure 4 had relatively similar XRD patterns, and only metallic Al and Bi were detected. The intensity of the Bi-rich phases was the only observation that distinguished the patterns from one another, which was expected due to the different amounts of Bi present in the composites. The peak intensity increased with higher wt% Bi present in the composite. However, it is important to note that the peak intensity is also influenced by the composite’s particle size and degree of crystallinity.

Figure 4 further exhibits no new phases in either one of the Al composites; the only detected phases were Al and Bi, which suggests that no intermetallic phases occurred between Al and Bi during the mechanochemical activation. This may be due to the low solid solubility of Bi in the Al matrix stated earlier [85]. The absence of the Zn-rich phase may jointly be ascribed to the low content of Zn in the composite, i.e., ≤5 wt%, and the possible dissolution of Zn in the Al phase as described by Zhang et al. [86]. In addition, XRD analysis of Al-Zn composites containing ≥15 wt% Zn performed by Bayraktar et al. indicated a single-Al ɑ-phase (solid solution) [87].

### 3.3. Hydrolysis of Ternary Al-Bi-Zn Composites

**Figure 5:**
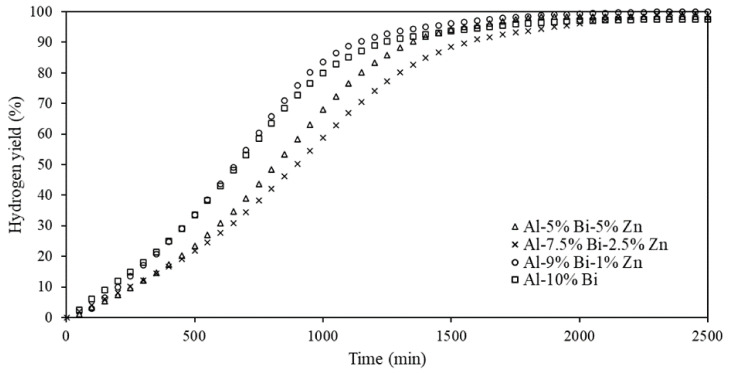
Hydrogen yields of Al-Bi and Al-Bi-Zn composites.

Ternary composites (see Table 1) were hydrolyzed in pure water to explore the synergetic effects of Bi and Zn on Al during hydrolysis. Figure 5 exhibits the hydrogen yields of the ternary Al-Bi-Zn composites. Composite Al-10% Bi is included in Figure 5 and serves as a reference to signify the effect of the various amounts of Bi and Zn on the composite reactivity. No standard deviations for experimental results are included in Figure 5 to avoid cluttering of the presented data. The maximum standard deviation did not exceed <3.8%.

Figure 5 shows that there were some slight differences in the curve shapes. The investigated composites had hydrogen yields of 97.4–99.9%. Composite Al-9% Bi-1% Zn had the highest hydrogen yield of 99.9%, and the reaction was completed after approximately 2250 s. The addition of 1 wt% Zn did, however, accelerate the hydrolysis reaction when compared to the Al-10% Bi reference curve.

The addition of >1 wt% Zn as a composite constituent increased the reactivity of the composites (when compared to Al-10% Bi) slightly while slowing the reaction rate. For instance, Al-10% Bi had a hydrogen yield of 97.4% and a reaction period of 1993s, while Al-7.5% Bi-2.5% Zn had a hydrogen yield of 99.5% and a reaction period of 2300s. A study by Du Preez et al. (2017) showed that a decrease in Bi addition resulted in an appreciable decrease in hydrogen yield % [11]. Considering that the main objective of this study was to delay the hydrogen release rate, it is clear from Figure 5 that the addition of Zn (>1 wt%) promoted a slower hydrogen release rate. The slightly elevated hydrogen yields, caused by the addition of Zn, can be ascribed to the promotion of pitting corrosion. A study carried out by Kireche et al. (2014) showed that the addition of Zn caused the pitting corrosion potential to take on more electronegative values, therefore promoting the formation of corrosion pits [88]. Furthermore, the incorporation of Zn can enhance the formation of cracks during the hydrolysis reaction due to volume expansion, which increases the active area between Al and water during the hydrolysis reaction [86].

It is, however, evident that the larger addition of Zn, and the subsequent decrease in Bi, did not show a linear change in the composite hydrolysis reactivity. For instance, 1 wt% Zn addition accelerated the hydrolysis activity (when compared to the reference case). However, increasing the Zn content to 2.5 wt% yielded a composite with milder activity than the composite containing 5 wt% Zn. It is therefore considered that a degree of synergy exists between the Bi and Zn composites, which may possibly be jointly ascribed to physical changes occurring during mechanochemical activation and/or the effect of the Bi/Zn ratio on the composite hydrolysis activity. It is, however, proposed that future research should be carried out to determine the governing effect Zn has on Al-Bi-Zn composites, morphological or reactiveness.

Table 2 was compiled to compare the results presented in Figure 5 to similar composites reported elsewhere. A recent comprehensive review summarized numerous ternary composites intended for on-demand hydrogen generation via hydrolysis [81]. Therefore, mainly Al-Bi-Zn composites reported in the public, peer-reviewed domain are reported here.

The composites shown in Table 2 suggest that the composites (Composites 1–3) prepared in this study had high hydrogen yields, whereas composites prepared via gas atomization yielded Composites 4–7 with the mildest hydrogen generation rates, considering that >92% hydrogen yields were achieved after >280 min. However, these composites had to be hydrolyzed at 50 °C. Composites 8 and 9 did not achieve appreciable hydrogen yields. Nevertheless, Table 2 shows that Al-Bi-Zn composites are suitable for mild hydrogen generation and do, however, require more investigation to obtain composites with high hydrogen yields and prolonged reaction times (similar to Composites 4–7) when hydrolyzed under ambient conditions.

### 3.4. Effects of Mass Ratio on the Hydrolysis Reaction

**Figure 6:**
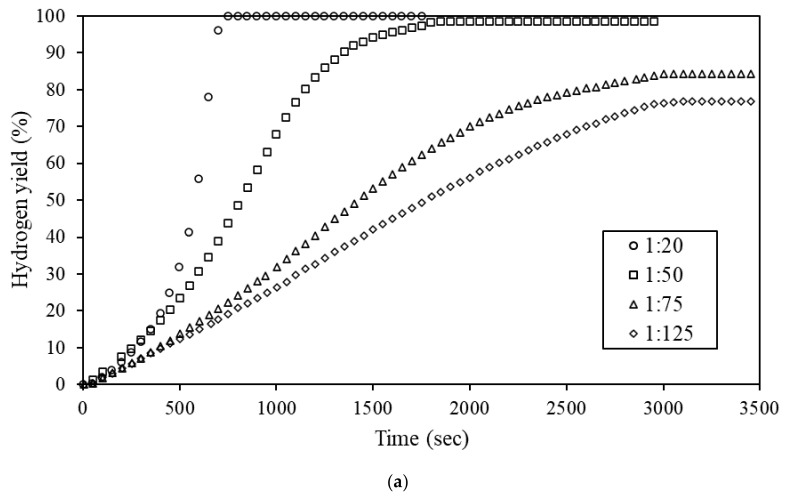

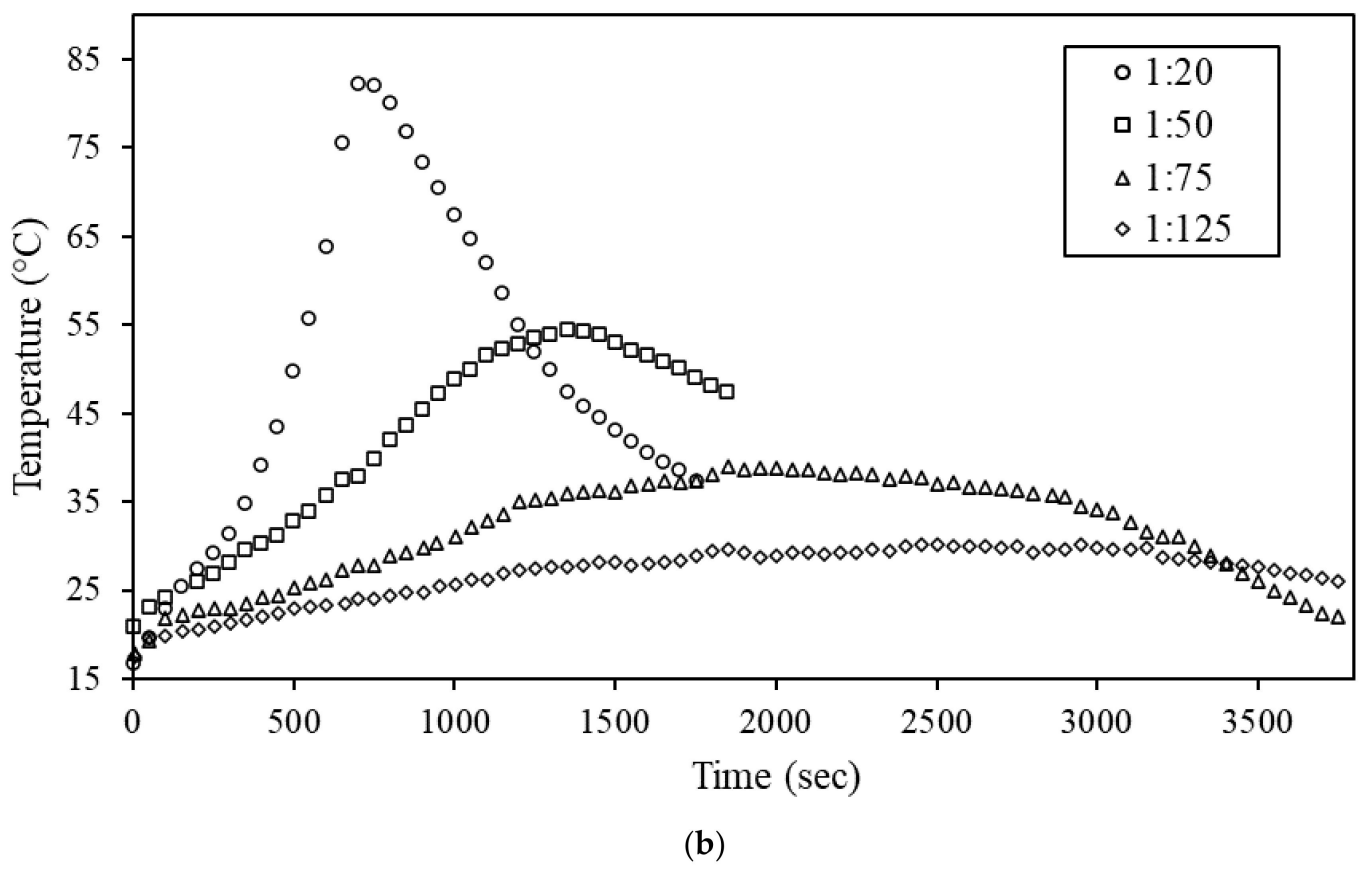
Effect of mass ratio on hydrogen yield of Al-5% Bi-5% Zn (**a**) and the change in reaction temperature during hydrolysis (**b**).

The Al-5% Bi-5% Zn composite was chosen to investigate the effects of mass ratio (defined as 1 g of Al powder per volume (mL) of deionized water) on the composite reactivity. This composite was selected as it presented a reactivity between the fast-reacting Al-9% Bi-1% Zn and slower-reacting Al-7.5% Bi-2.5% Zn composite. Figure 6 shows the effect of different mass ratios on hydrogen yield (a) and reaction temperature (b). The mass ratio ranged from 1:20 to 1:125.

It is evident from Figure 6a that the increase in mass ratio from 1:20 to 1:125 caused an appreciable decrease in hydrogen yield. Hydrogen yield of >99.9 and 76.9% was obtained for the 1:20 and 1:125 mass ratio hydrolysis reactions, respectively. It is known that the Al hydrolysis reaction is dependent on temperature, meaning the lower hydrogen yield was caused by the decrease in reaction temperature. In turn, the reaction temperature is dependent on the volume of water used during hydrolysis. In a larger mass ratio, the volume of water present during hydrolysis is greater, causing the hydrolysis reaction temperature to lower as the in situ generated heat (ΔH = −444.4 kJ.mol^−1^) [12] disperses away from the immediate reaction sites. The in situ generated heat is responsible for catalyzing the hydrolysis reaction.

Figure 6b shows that the reaction temperature decreases with an increasing mass ratio, subsequently resulting in a slower hydrogen generation rate and a decrease in hydrogen yield. As expected, all the Al composites evaluated in this study showed a similar trend. The reaction temperature of the 1:20 mass ratio hydrolysis reaction increased by approximately 64 ± 5.7 °C, while the temperature of the 1:125 mass ratio reaction increased by 11.8 ± 3.0 °C. A similar observation was made by Du Preez et al. (2017) during the hydrolysis of Al-In-Bi-Sn and Al-Bi-In composites [11,12].

**Figure 7:**
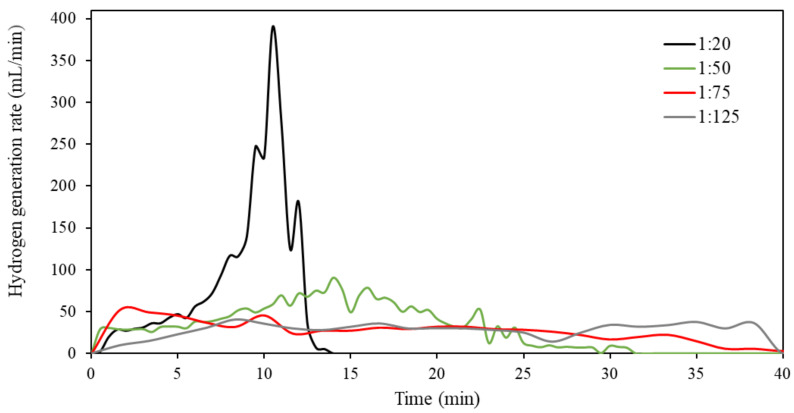
Hydrogen generation rates of Al-5% Bi-5% Zn composite hydrolyzed at various mass ratios.

The effect of mass ratio on the hydrogen generation rate of the Al-5% Bi-5% Zn composite was determined and is presented in Figure 7. It can be seen from Figure 7 that the peak hydrogen generation rate at 1:20 mass ratio was 390.4 mL/min, which was significantly higher compared to the larger mass ratios of 1:50 (90.7 mL/min), 1:75 (52.5 mL/min), and 1:125 (37.8 mL/min). Hence, the hydrolysis reaction of 1:20 mass ratio was complete after about 13 min, which was the same time when the 1:50 mass ratio achieved its maximum hydrogen generation rate.

Both 1:75 and 1:125 mass ratios had very similar hydrogen generation curves, which were flatter than the curves observed for 1:20 and 1:50 mass ratios. The flatter hydrogen generation rate curve indicates a systematic generation of hydrogen, which is ideal for hydrogen-consuming applications such as PEMFC. Though a flatter curve was obtained, one has to consider that the hydrogen yields of the 1:75 and 1:125 hydrolysis reactions were significantly lower than the 1:50 reaction. Therefore, the optimal reaction conditions have to be carefully selected for a particular application. For instance, sacrificing hydrogen yield % for a mild hydrogen generation rate during simple PEMFC application or practically accommodating a higher hydrogen release rate via process design while retaining a high hydrogen yield.

### 3.5. Analysis of Hydrolysis Residues

**Figure 8:**
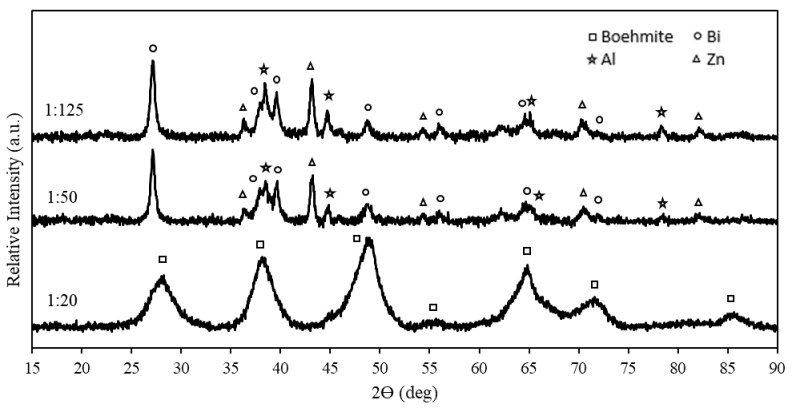
Hydrolysis residues of ternary composite Al-5% Bi-5% Zn at various mass ratios.

The hydrolysis residues of Al-5% Bi-5% Zn at different mass ratios (1:20, 1:50, and 1:125) were characterized via XRD (Figure 8). It is evident from Figure 8 that the XRD pattern of 1:20 mass ratio only consisted of large boehmite (AlOOH), which suggests that the entire Al constituency was hydrolyzed. Al peaks were only observed in the 1:125 and 1:50 residues; this is indicative that some Al did not participate in the hydrolysis reaction. The presence of Al-hydroxide peaks was not evident for 1:50 and 1:125 hydrolysis reactions; it is, however, likely that boehmite peaks were masked by overlapping Al and Bi peaks. A similar observation was made by Du Preez et al. (2017) [11]. The Bi and Zn peaks exhibited in the 1:50 and 1:125 residues indicate that Bi and Zn were present as segregated phases and that these elements were not oxidized.

The observed Zn peaks in Figure 8 substantiate that the absence of Zn peaks in Figure 4 was caused by its inclusion as part of the Al phases. The conversion of Al to AlOOH revealed the underlying Zn peaks, which are visible in Figure 8.

### 3.6. Effects of Water Quality on Hydrolysis

**Figure 9:**
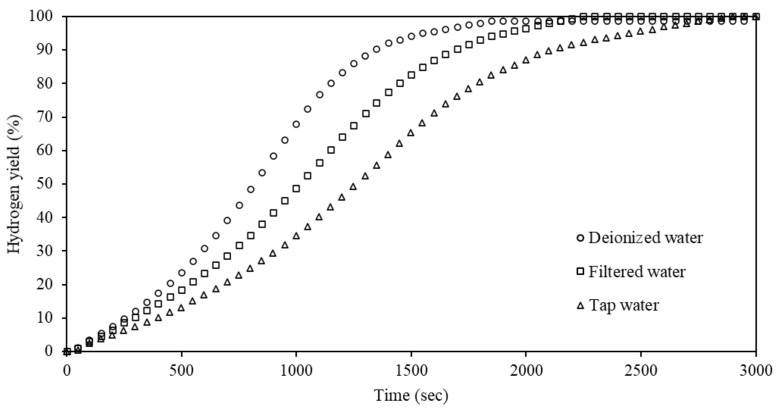
Hydrogen yield curves of Al-5% Bi-5% Zn hydrolyzed using different water qualities.

Considering that in many cases deionized water will likely not be accessible for hydrolysis, it was considered to evaluate the effect of water quality on the hydrolysis of Al-5% Bi-5% Zn. Hydrolysis was performed in three water qualities, i.e., deionized, filtered, and tap water. Figure 9 shows these results.

According to Figure 9, the employment of different water qualities did not have an appreciable effect on the final hydrogen yield of Al-5% Bi-5% Zn. The hydrogen yields in filtered water and tap water were similar (99.9%) and slightly higher than that in deionized water (98.5%). However, the water quality had an appreciable effect on the hydrolysis kinetics of the Al-5% Bi-5% Zn composite. Hydrolysis performed in deionized water had the fastest hydrolysis kinetics, while hydrolysis in filtered and tap water was milder.

Considering the various hydrolysis kinetics observed in Figure 9, tap water would be the ideal water quality for PEMFC applications due to the aforementioned slow hydrolysis kinetics. Additionally, the use of tap water foregoes pre-treatment procedures, i.e., deionization and filtration. It is not clear how the presence of ions typically present in tap water affects the hydrolysis kinetics without adversely affecting the hydrogen yield. It is proposed that these ions delay the rate of Al to Al^3+^ oxidation without forming permanent bonds with the Al ions. This phenomenon does, however, require further investigation.

**Figure 10:**
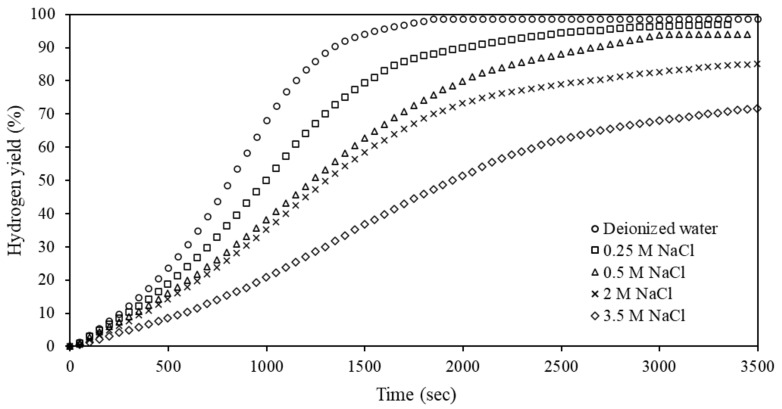
Hydrogen yield of Al-5% Bi-5% Zn composite reacting with different concentrations of NaCl solutions at room temperature.

To further investigate the effect of water quality, NaCl solutions of various concentrations were prepared. Figure 10 presents these results. The hydrolysis of Al-5% Bi-5% Zn in deionized water was included as a reference.

It is evident from Figure 10 that the hydrolysis reaction rate of the Al-5% Bi-5% Zn composite reacting in deionized water was higher when compared to the NaCl solutions. Furthermore, both the hydrolysis reaction rate and the hydrogen yield decreased as the NaCl concentration increased. The hydrogen yield decreased from 97.5 to 75.9% with an increase in NaCl concentration of 0.25–3.5 M. The observed decrease in reaction rate and hydrogen yield was unexpected considering that the inclusion of an electrolyte should increase the conductivity of the hydrolysis solution [1,75,90]. A recent study by Wang et al. (2021) observed a similar trend [50]. Currently, this phenomenon is unexplained and requires further investigation.

### 3.7. Proposed Applications

When selecting a material for on-demand hydrogen generation, several factors are considered, e.g., material cost, hydrogen generation capacity, physical properties (gravimetric density, kg/m^3^), and chemical properties (stability in ambient atmosphere). For instance, the hydrolysis of lithium (Li) can yield 1.76 L of hydrogen per g of Li, as opposed to 1.36 L per g of Al. Li is, however, reactive with air and necessitates specialized storage procedures.

Hydrogen generated by the hydrolysis of the Al composites prepared in this study is suitable for PEMFC applications. More so, the specific application will determine the ideal hydrogen generation rates, e.g., a higher hydrogen supply will be required during device start-up, while a lower supply is required during device idling. In the case of a PEMFC power application, and in general, the systematic release of hydrogen is associated with milder hydrolysis reaction temperatures and subsequent lower hydrogen yields. For example, the hydrolysis of 1 g of Al-5% Bi-5% Zn at a 1:20 mass ratio achieved a hydrogen yield of close to 100%, a hydrogen generation peak of approximately 390.4 mL/min, as well as a 64 °C reaction temperature increase. However, such extreme conditions will likely result in complicated PEMFC operations. Nevertheless, at a 1:50 mass ratio, the reaction temperature increased by 33.6 °C, and a peak hydrogen generation rate of 90.7 mL/min was achieved while achieving a 98.5% hydrogen yield. Therefore, the hydrolysis of a specific amount of the Al-5 wt% Bi-5 wt% Zn at a 1:50 mass ratio may supply a steady stream of hydrogen to a PEMFC. The size of the PEMFC will determine the amount of Al composite required for hydrolysis.

Though current PEMFC technologies and such technologies consuming on-demand generated hydrogen require improvement, the proposed utilization of on-demand hydrogen generation by the hydrolysis of Al is suitable in certain sectors. These sectors are, however, mainly limited to low power (<1 kW) devices. For instance, according to the Russian Skolkovo Foundation, the portable charging devices market is valued at USD 34 bn [91]. Considering the high hydrogen yields achieved from the investigated composites (97.4–99.9%), an estimated 2.2 kWh kg^−1^ Al of specific electric energy can be produced through a PEMFC. Furthermore, generating hydrogen through Al hydrolysis is advantageous as it supplies pure on-demand hydrogen, which lessens the difficulty of hydrogen storage, purification, and transport [92].

Approximately 25% electrical energy and 75% thermal energy can be recovered from the accessible energy of Al hydrolysis-based energy technology [81]. The thermal energy generated through Al hydrolysis can also be utilized in alternative markets. For instance, Godart and Hart (2020) showed that the thermal energy generated during hydrolysis can be utilized to desalinate seawater via reverse osmosis by expanding a piston that pushes seawater against a semi-permeable membrane, resulting in drinkable water permeating [93]. According to Shkolnikov et al. (2011), thermal energy is underutilized, which reduces the overall energy efficiency of the Al hydrolysis energy system [94]. It is thus proposed that alternative uses of the thermal energy generated during hydrolysis be utilized to improve the overall efficiency of the hydrolysis of Al. It is also noted here that reaction heat may pose an operational issue if not managed properly. Heat management should be considered during the design-phase of a proposed application.

## 4. Conclusions

A ball milling method was employed for the formulation of mechanochemical Al composites consisting of 90 wt% Al and various amounts of Bi and Zn as activation metals (10 wt% total). In some cases, Zn-containing composite distribution and particle size reduction did not occur, suggesting the inability of Zn to promote Al’s structural degradation. However, composites that did undergo a visually observable size reduction had a relatively uniform distribution of Bi and Zn, as confirmed by surface SEM-EDS. The presence of Bi and Zn throughout Al particles allowed the formation of micro-galvanic cells between anodic Al and cathodic Bi/Zn. XRD analysis indicated that Al and Bi are the major phases present in the composites after mechanochemical activation. Only after hydrolysis was Zn detected, which indicates that Zn was solubilized in the Al phase.

All reactive Al-Bi-Zn composites had hydrogen yields of >98%interaction, which were marginally higher than the Al-10% Bi composite (i.e., 97.4 %). The addition of Zn beyond 1 wt% slowed the hydrolysis reaction kinetics, and by performing the hydrolysis reaction in tap water, the kinetics could be slower further. In addition, by increasing the mass ratio, the hydrogen generation rate could be reduced at the cost of lowering the hydrogen yield. The utilization of NaCl solutions caused the hydrogen yield and hydrolysis reaction rate to decrease.

## Figures and Tables

**Table 1:** Ternary Al composite (Al-Bi-Zn) compositions (wt%).

Composites	Activation Metals (wt%)	
	Bi	Zn
Al-5% Bi-5% Zn	5	5
Al-7.5% Bi-2.5% Zn	7.5	2.5
Al-2.5% Bi-7.5% Zn	2.5	7.5
Al-9% Bi-1% Zn	9	1
Al-1% Bi-9% Zn	1	9
Al-10% Bi	10	0
Al-2.5% Bi-2.5% Zn	2.5	2.5

**Table 2:** Comparison of Al-Bi-Zn composites.

Composite	Activation Metal		Yield (% or mL/g)	Reaction Time (s or Min)	Ref.
	Bi	Zn			
1	9	1	99.9%	2300 s	This work
2	7.5	2.5	99.5%	2400 s	
3	5	5	98.5%	1850 s	
4 *	12	7	98%	280 min	[50]
5 *	11	8	94%	300 min ^#^	
6 *	10	9	94%	300 min ^#^	
7 *	9	10	92%	320 min ^#^	
8	10	10	830 mL/g	5 min	[72]
9	10 (Sn)	10	680 mL/g	14 min	[89]

* composite prepared via gas atomization, hydrolysis performed at 50 °C. ^#^ values determined from figure

## Data Availability

Not applicable.
